# A model for delivering subspecialty pediatric surgical care in low- and middle-income countries: one organization’s early experience

**DOI:** 10.1186/s40064-015-1528-z

**Published:** 2015-11-27

**Authors:** Tyler K. Merceron, Ligia Figueroa, Quentin E. Eichbaum

**Affiliations:** Vanderbilt University School of Medicine, Nashville, TN USA; Centro Quirurgico Pediatrico Moore, Guatemala City, Guatemala; Vanderbilt Institute for Global Health, Nashville, TN USA; Department of Pathology, Microbiology and Immunology, Vanderbilt University Medical Center, Nashville, TN USA

**Keywords:** Global surgery, Pediatric surgery, Guatemala, Moore Pediatric Surgery Center

## Abstract

Delivery of humanitarian global surgical aid to low-middle income countries (LMICs) often occurs as a “fly-in, fly-out” marathon of operations. Unfortunately, the sustainability and efficacy of these missions remain questionable because they are difficult to reproduce and they have limited ability to provide peri-operative care. The goal of this project was to describe the Moore Pediatric Surgery Center (MPSC) in Guatemala City as an alternative model that provides a centralized structure to the interaction between surgical providers and patients in the operative and peri-operative periods. We also describe the Center’s patient population and present feedback from surgical teams visiting the MPSC. A retrospective chart review was performed to quantify the number of patients, procedures, and post-operative complications at the MPSC between January 2011 and December 2014. We also performed a cross-sectional sociodemographic survey of MPSC patients and conducted a satisfaction survey of patients and surgical team members visiting the Center. Since 2011, the MPSC has hosted 42 surgical teams representing 7 different specialties. During its first four years, the surgery center hospital performed 2260 operations with a 1.07 % peri-operative complication rate and 0 % peri-operative mortality rate. All surgeries were performed free-of-charge to children from low-income households. Furthermore, the MPSC was rated highly among visiting team members (range 4.5–6 on a 7-point Likert scale) for quality metrics including organization, physical space, and collaboration with local staff. The MPSC represents a model for delivering multi-specialty surgical aid in low- and middle-income countries by providing modern surgical facilities with quality-assured post-operative care for the treatment of childhood surgical diseases.

## Background

Medical aid to low- and middle-income countries has focused primarily on infectious diseases, malnutrition, and maternal-neonatal care. In contrast, global surgical aid programs are not yet well established. Surgery has even been referred to as “the neglected stepchild of global public health [efforts]” (Farmer and Kim 2008). Over the last decade, however, surgical diseases have gained attention as an important component of the public health paradigm (Ivers et al. 2008). By some estimates, the global burden of surgical disease lies between 11 and 28 % of the total world disability-adjusted life years (DALYs) (Debas et al. 2006; Shrime et al. 2014). These diseases span the scope of every surgical specialty, necessitating innovative models that provide comprehensive, multi-specialty surgical care.

The most common form of global surgical aid is the short-term medical mission. These missions involve a team of physicians, nurses and staff volunteering their time, equipment and services. Partnerships between host and donor countries often arise from interpersonal contacts to coordinate operative space and peri-operative care (Baran and Tiftikcioglu 2007; Mainthia et al. 2009; Merrell et al. 2007). While these efforts can deliver a large volume of high quality surgery, they are limited in that they are often the result of non-reproducible ad hoc connections between physicians from different countries. Moreover, limited resources and time often lead to restrictions in operative scope and an inability to ensure the post-operative care of the patients.

This paper describes the work of the Shalom Foundation’s (SF) surgical initiative in Guatemala. SF is a non-profit organization that is dedicated to providing educational opportunities, food and clean water, shelter, and medical assistance to underprivileged children and their families in Guatemala. We focus specifically on the Moore Pediatric Surgery Center (MPSC), SF’s independent surgical hospital. Built in 2011, we believe the MPSC is a unique model for the delivery of global surgical aid in that it provides a centralized organizational structure though which visiting surgeons, local providers and patients can interact to provide optimal surgical care. The aim of this work is to describe the MPSC model of care, review the operative records of SF-directed surgical missions in Guatemala before and after construction of the MPSC, describe the MPSC patient population, and review feedback from patients and healthcare professionals interfacing at the MPSC.

### How the Moore Pediatric Surgery Center model works

As a structure, the MPSC has 12,000 square feet of space and functions as an independent surgical hospital. It has 3 modern operating rooms, 4 pre-operative holding beds, 21 post-operative recovery beds, a nursing station, and a stocked pharmacy. While visiting surgical teams bring some of the medications needed for their specific peri-operative protocols, the pharmacy is equipped with the anesthetics, analgesics, and antibiotics necessary for surgery and the treatment of potential complications. The hospital also has a conference room, administrative offices, waiting areas, laundry facilities, a kitchen, residents’ quarters, 24-hour security and wireless Internet capacity.

**Fig. 1:**
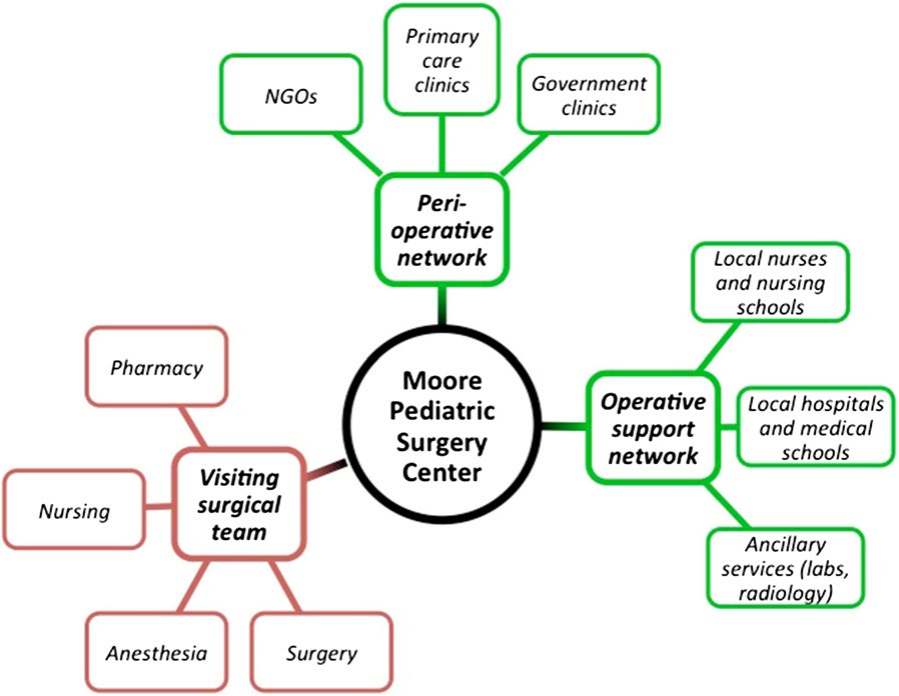
Schematic map showing connections of providers interacting with the MPSC. The MPSC (*black*) serves as the intersection between a variety of providers from the United States (*red*, “visiting surgical team”) and Guatemala (*green*, “peri-operative network” and “operative support network”)

The MPSC thus provides an independent space and organizational structure for visiting surgeons, local providers and patients to coordinate operative and peri-operative care (Fig. 1). The MPSC is owned by SF and managed by a local Guatemalan staff of administrators, physicians, nurses, and ancillary service personnel. This staff provides consistency in the practice that occurs at the MPSC as various visiting surgical teams rotate through.

**Fig. 2:**
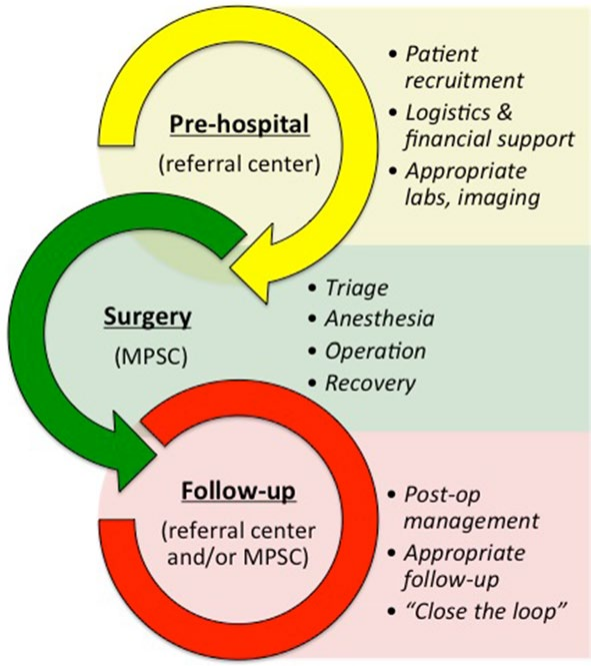
Normal flow of a surgical tour at the MPSC. During the pre-hospital phase (*yellow*), patients are referred for evaluation and surgical teams are recruited. During the surgery phase (*green*), patients are triaged based on surgical need, risk, and resource availability and the operations are carried out. Finally, during the follow-up phase (*red*), patients are evaluated either by the MPSC or a member of the peri-operative network in their hometown and post-operative complications are managed

A typical mission proceeds in three phases: pre-hospital, surgery and follow-up (Fig. 2). Before a surgical tour can take place, the SF staff coordinates with teams from various academic and private surgical groups to plan dates and the types of procedures to be done during the ‘pre-hospital phase’. The hospital is then booked for the period of time during which surgery will take place (often 1 week). During the pre-hospital phase, surgical teams will work with the SF/MPSC staff to coordinate what specialty supplies and/or medications the team needs to bring with them, as well as lodging, travel, and all other necessary arrangements. It is also during this time that patients are recruited for surgery.

Once the team arrives in Guatemala City, the ‘surgery phase’ begins. On the first day, the team is brought to the MPSC for orientation with the Guatemalan staff. The following day is “triage day”, with up to 100 patients being screened by the surgeons and anesthesiologists. The triage process takes into account all of the routine evaluation for surgery (e.g., safety for surgery, safety for anesthesia, patient co-morbidities) as well as the available human and material resources, a case-by-case assessment of disease burden and severity, and available time. Once the patients have been triaged, teams work for the rest of the week to perform all operations that need to be done, turning over an average of 10–20 cases per day depending on the complexity of the procedures. After surgery, the patients are either kept in house or in a shelter close by.

On the last day of the mission, the ‘follow-up phase’ begins. All patients are re-evaluated to make sure they are doing well in the immediate post-operative period. It is at this time that an additional follow-up date may be scheduled, should that be deemed necessary by the surgical team. In this way, patients are able to benefit from complete care before, during, and after their operation.

## Methods

A retrospective study was performed on operations occurring between January 2011 and December 2014. Data collected included number of patients screened for surgery, number of patients receiving operations, number of patients receiving other services, and hometown. Two additional prospective studies were conducted during surgical visits to the MPSC. The first was an exit questionnaire administered to patients prior to discharge. The objective of this questionnaire was to identify and describe the patient population based on various sociodemographic characteristics including family size, number of dependents, household income, and patient satisfaction. We also asked visiting surgical team members to complete a survey evaluating the MPSC staff and other quality metrics, including collaboration with local staff, communication, organization, available technology, the post-anesthesia care unit, and cleanliness. The Vanderbilt University Medical Center institutional review board approved this study and its tools, and informed consent was obtained from all study participants.

The data were collected and managed using REDCap electronic data capture tools hosted at Vanderbilt University Medical Center. REDCap (Research Electronic Data Capture) is a secure, web-based application designed to support data capture for research studies, providing (1) an intuitive interface for validated data entry; (2) audit trails for tracking data manipulation and export procedures; (3) automated export procedures for seamless data downloads to common statistical packages; and (4) procedures for importing data from external sources (Harris et al. 2009).

## Results

### Retrospective review of operations

**Fig. 3:**
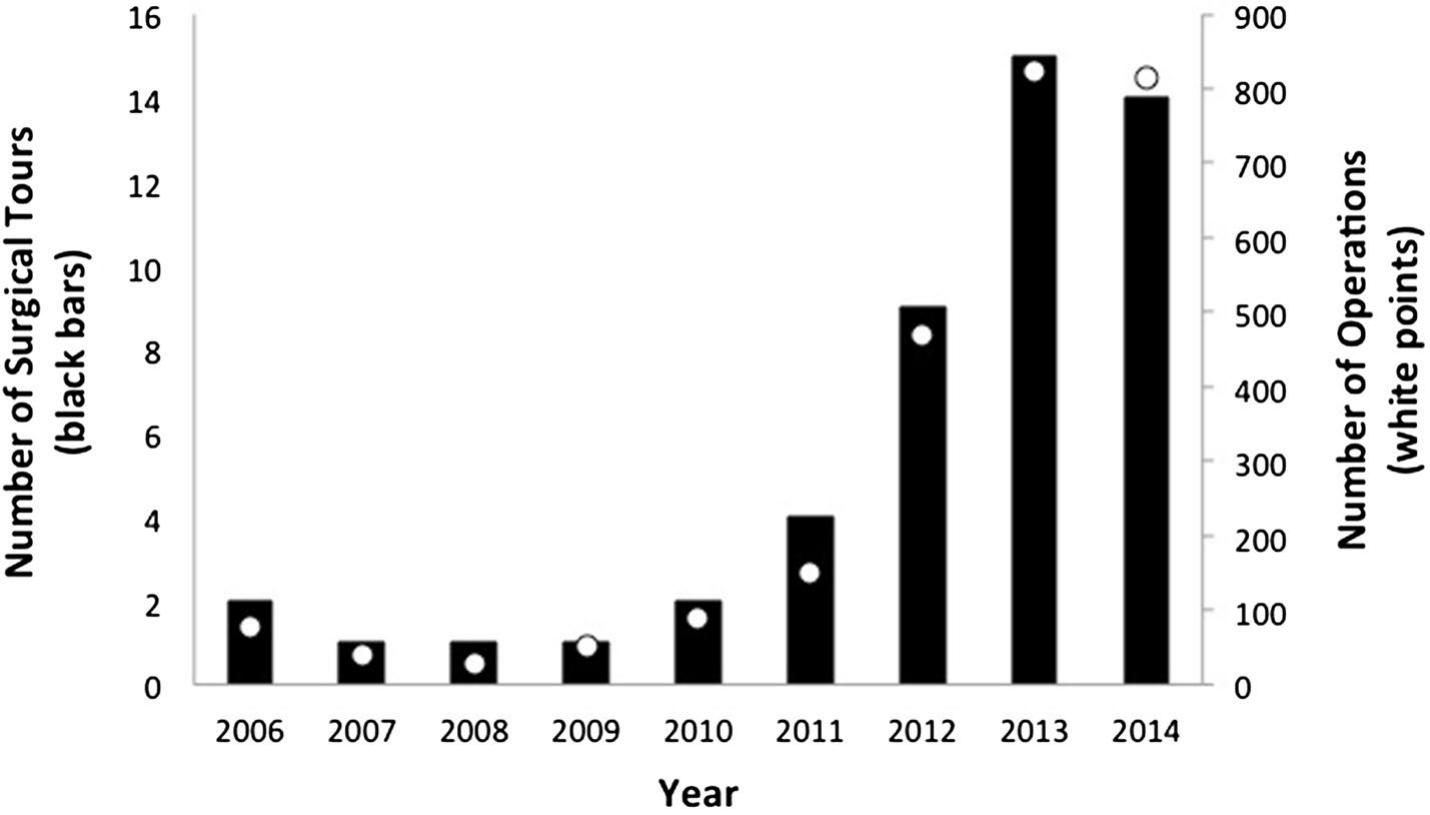
Graph showing the number of surgical tours and operations coordinated by SF between 2006 and 2014. Note the significant increase in surgeries beginning in 2011, after the opening of the MPSC

Between 2006 and 2014, SF sponsored 49 pediatric surgical tours and 2542 operations in Guatemala (Fig. 3). In the 5 years preceding construction of the MPSC (2006–2010), 7 surgical tours were completed yielding a total of 282 operations (14.3 % and 11.1 % of the total tours and operations between 2006–2014). This is in contrast to the 42 surgical tours and 2260 operations that occured in the 4 years following construction (85.7 % and 88.9 % of the total tours and operations between 2006–2014). The MPSC began systematically collecting more data from patients beginning in 2013, including hometown and the receipt of other non-surgical care. In the period between January 2013 and April 2014, 635 (56.5 %) patients were from within the Guatemala City limits, and 489 (43.5 %) were referred from other parts of the country. 351 (31.2 %) patients received other support, including diagnostic labs, imaging, pathologic evaluation, transportation and/or shelter during this time period.

**Fig. 4:**
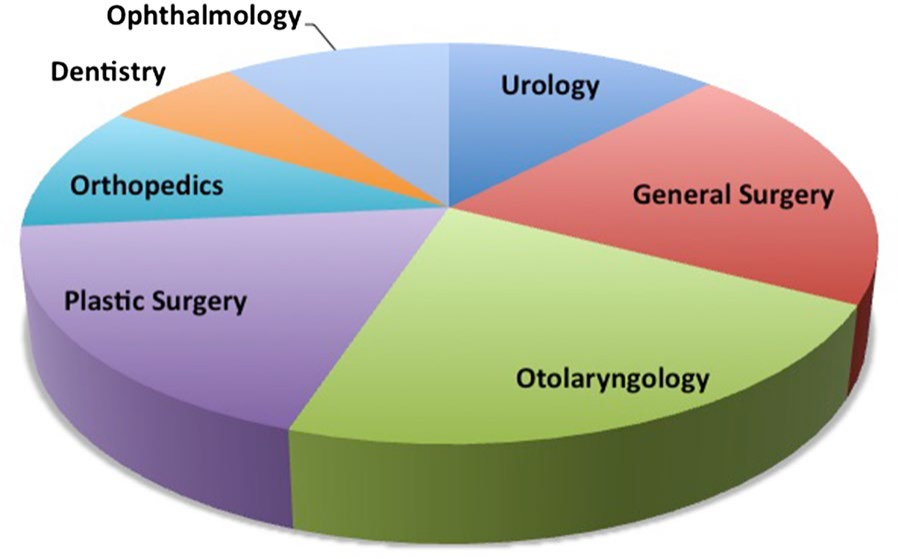
Breakdown of specialty services hosted by SF from 2006 to 2014. Otolaryngology (22.4 %), general surgery (20.4 %), plastic surgery (18.4 %), urology (12.2 %), orthopedics (10.2 %), ophthalmology (10.2 %), dentistry (6.2 %)

Specialties have included general surgery, urology, plastic surgery, dentistry, otolaryngology, ophthalmology, and orthopedics (Fig. 4). The most common procedures varied depending on the specialty performing the mission. General surgeons performed hernia repairs, splenectomies, cholecystectomies, appendectomies, and mass/cyst removals. Urologists performed hypospadias repair, cryptorchidism repair, and phimosis repair. Plastic surgeons performed scar revisions, cyst/mass excision, and microtia repair. Dentists attended to dental caries, fillings, extractions and root canals. Otolaryngologists performed cleft lip/palate repairs, tonsillectomies, adenoidectomies, thyroglossal duct cyst removals, and tympanoplasties. Ophthalmologists performed strabismus repair, ptosis repair and chalazion removal. Orthopedic surgeons performed repairs of clubfoot, congenital dislocation of the hip, nerve injuries of the hand, polydactyly and syndactyly.

Overall, the complication rate has been 1.07 % for all cases performed. These complications have included surgical wound site infection, bleeding/hematoma formation and wound dehiscence. Because Guatemalan physicians continue to staff the MPSC when the visiting surgical team leaves, patients were able to return to the center to have their complications attended to. All patients with wound site infections were given the appropriate course of antibiotics and/or appropriately drained, and MPSC physicians were capable of attending to all incidents of bleeding and wound dehiscence. The peri-operative mortality rate over the time course reviewed was 0 %.

### Patient sociodemographic and satisfaction questionnaire

The MPSC only treats children who do not have alternative means of surgical treatment. All families surveyed made less than US $533.33 a month, with two-thirds making less than US $266.67 monthly (Table 1). The average household size was between 6 and 7 people, with an average of 3–4 children per household. Finally, 100 % of the patients surveyed rated their care as “good (6)” or “excellent (7)” on a 7-point Likert scale.

**Table 1:** Sociodemographic for patients treated at the MPSC

Metric	Value
Families making <Q 2000^a^ per month	66 %
Families making Q 2000–4000^a^ per month	34 %
Average persons per household	6.4
Average children per household	3.3

^a^Reference: US $1.00 = Q 7.50; average annual Guatemalan income = Q 2856.25

### Visiting surgical team satisfaction survey

**Fig. 5:**
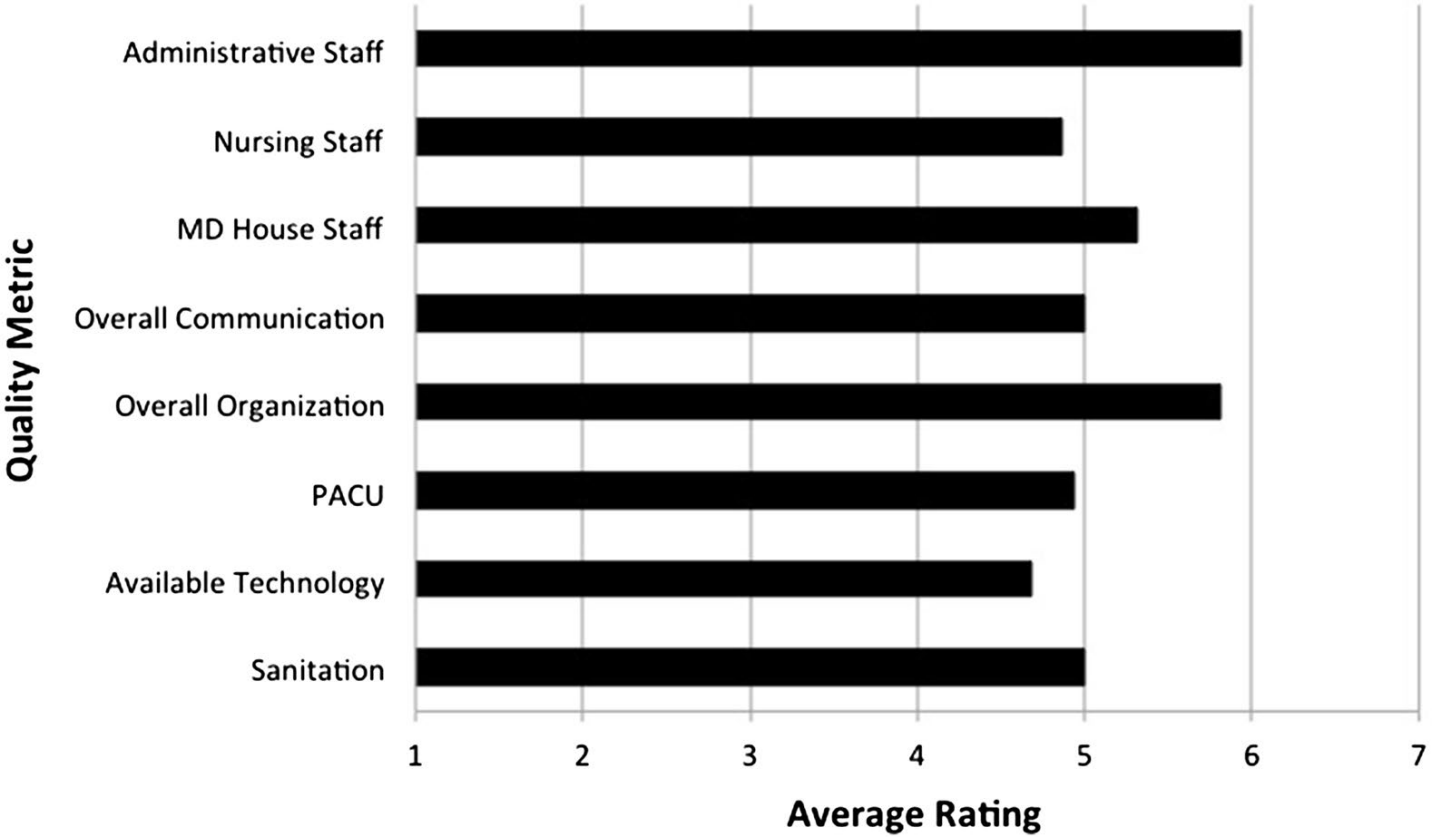
Average ratings for the MPSC for various quality metrics. Likert scale: *1* (significantly below average), *2* (below average), *3* (slightly below average), *4* (average), *5* (slightly above average), *6* (above average), *7* (significantly above average)

Mean quality ratings for collaborative efforts with local administrative and medical house staff were consistently within the “slightly above average (5)” to “above average (6)” range, with nursing staff falling just below the “slightly above average (5)” mark on a 7-point Likert scale. Overall communication and organization of the MPSC ranged between “slightly above average (5)” to “above average (6)”. The post anesthesia care unit (PACU), available technology and sanitation were rated with in the “average (4)” to “slightly above average (5)” range (Fig. 5).

## Discussion

Guatemala is ranked 133rd of the 187 countries and territories listed in the United Nations’ 2013 Human Development Index. In Guatemala, children under 18 years of age represent approximately 50 % of the total population, and 51 % of people live below the international poverty line. Guatemala also has the highest level of chronic malnutrition in Central and South America (Guatemala 2012). With 9.3 physicians per 10,000 and an annual per capita expenditure on health of US $259, Guatemala has one of the poorest healthcare systems in the Latin America (Observatory 2012). Underfunded and concentrated in the more urban parts of the country, the system is inaccessible to a large number of Guatemalans, particularly children. For most, surgery is never an option. It is within this context that novel methods for delivering quality global surgical care are needed.

Contemporary surgical aid is often performed as a “fly-in, fly-out” marathon of procedures and operations. While this strategy can deliver a high volume and skill level of surgery, it is problematic for various reasons, including: (1) low capacity to provide quality post-operative care; (2) high cost of purchasing and importing surgical equipment, anesthesia and mediations; (3) frequently poor conditions for surgery; (4) and cultural discrepancies (i.e. the notion of obtaining informed consent) (Crompton et al. 2010; Dunser et al. 2006; Isaacson et al. 2010). Thus, a successful global surgical aid program would be one that is well orchestrated and focused on the provision of safe and effective patient care by: (1) ensuring both a quality operation and the appropriate peri-operative and follow-up care; (2) providing a steady source of human and material resources in a manner that supports the local economy (i.e. utilizing local diagnostic laboratories and purchasing medications from local pharmacies); (3) operating in a dedicated space with modern technology and a reliable source of electricity and water; and (4) being foundationally integrated within the host country’s culture and infrastructure.

The MPSC aims to address these limitations by offering a “hybrid” model of global surgical aid. This model is novel in that it has a permanent local and administrative component (e.g., the hospital and its Guatemalan staff) and a rotating cadre of visiting surgical subspecialists. The MPSC provides an intersection via which American and Guatemalan teams can collaborate. In this way, visiting surgical teams can quickly and seamlessly integrate into the local healthcare landscape allowing for the complete care of the patient with no extra work required of the volunteer team. Centralization of the organizational structure also affords additional benefits, including: (1) ability to enlist a wide array of surgical specialists, (2) more efficient, coordinated recruitment of patients and surgeons, and (3) the ability to provide the appropriate peri-operative care to the patient under one roof. Our results show that this model can provide a sustained high volume of surgery from a variety of specialists with the appropriate level of follow-up and management of post-operative complications. Further, the construction of a dedicated surgical space dramatically increased the number of operations that could take place. If the current trend continues, we can expect that many more children requiring operations will be attended to, with the potential for more complex, multidisciplinary procedures (e.g., plastic and ENT surgeons working together for complicated craniofacial reconstructions). Furthermore, as surgical capacity increases, the opportunity for educational programs for local medical students and surgical residents will grow proportionally beyond current informal rotations.

With the increased capacity for surgery that the MPSC provides, a primary effort is currently being made by the staff to expand services to patients living outside of Guatemala City, as there is a large unmet need in the more remote regions of the country. These areas often have little or no access to medical care, much less a surgeon and an operating room. Because of this, it is important that the MPSC create strong partnerships with healthcare NGOs and government-run clinics (“Centros de Salud”) operating in the smaller villages throughout Guatemala. These relationships can constitute a robust network, with the rural clinic working in concert with the MPSC to provide complete peri-operative care to the patient. The MPSC would provide the surgery while the rural clinics would serve a dual role to refer patients and monitor the post-operative status of the patient.

Surgical mission trips often target patients from low socioeconomic backgrounds. The MPSC mission is no different. All of the patients treated by the hospital live around or below the international poverty line of US $1.25/day, with the majority of families living significantly below it. Furthermore, Guatemalan families are often large. The population we polled had an average of 3–4 dependents per household, meaning that a finite sum of money is spread very thin. Because these patients have no means of paying for their care, the MPSC relies on a time-share financial model where surgical teams invest a pre-set fee into the hospital. This fee covers the cost of operating the center for the team’s tenure, including the costs of equipment, supplies, medications, and staff. Combined with philanthropic support from American and Guatemalan sources, the MPSC is able to function as an independent entity with a reliable fund of capital and resources. This financial structure has resulted in the ability to provide free care to a needy population with a high level of patient satisfaction.

The final element required for a sustainable healthcare enterprise is ensuring the satisfaction of the providers (both visiting and local). The MPSC’s centralized structure allows the ability to collect quality improvement data and execute the changes needed for success. Overall, the MPSC was rated above average on all quality metrics analyzed. While these high marks are encouraging, there were some notable areas for improvement. The lowest rating was for the local nursing staff. Based on comments from the providers polled, Guatemalan nurses lacked certain skills that are commonplace among American nursing staff. Recognition of these differences in training and skills resulted in a more appropriate delegation of tasks between Guatemalan and American nurses, as well as an opportunity for teaching between the two groups. As time passes and new opportunities for improvement arise, the MPSC model is well suited to institute the changes required for the delivery of superior care.

In conclusion, the MPSC is a viable model for providing subspecialized pediatric surgical care in low- and middle-income countries. From our review of this hospital and its organizational structure, the center provides an array of surgical services for an underserved community of low-income, resource-limited children with a high level of care in the eyes of patients and providers. In the future, we expect this model can be expanded and integrated into the existing healthcare community by partnering with established clinics and governmental organizations.
